# Trauma-Focused Treatment in Early and Lifetime Psychosis: A Scoping Review

**DOI:** 10.1093/schbul/sbaf202

**Published:** 2026-08-20

**Authors:** Samantha E. Jankowski, Rémy Bennett, Bradley Tao, Yuval Neria, Lisa B. Dixon, Milton Wainberg, Annika Sweetland, Sapana R. Patel

**Affiliations:** 1 Department of Psychiatry, Columbia University Medical Center, New York, NY 10032, United States; 2 Department of Psychiatry, New York State Psychiatric Institute, New York, NY 10032, United States

**Keywords:** psychosis, trauma, PTSD, psychological treatment, scoping review

## Abstract

**Background and Hypothesis:**

Despite high rates of trauma exposure and posttraumatic stress disorder (PTSD) in individuals living with psychosis, there is limited knowledge regarding which trauma-focused treatments are most appropriate to address comorbid psychosis and PTSD, particularly in early psychosis, a clinically and developmentally distinct period.

**Study Design:**

This scoping review aims to understand the extent and type of evidence available to address PTSD symptoms at different stages of psychosis (ie, early psychosis versus lifetime diagnosis). Regarding early psychosis, our review considers studies involving individuals who are within 5 years of illness onset or are enrolled in early intervention services for first episode psychosis. The review was pre-registered in Open Science Framework. Three databases (PubMed, PsycINFO, Web of Science) were searched using specific keywords related to psychosis, trauma, and treatment. PRISMA-ScR guidelines for scoping reviews were followed.

**Study Results:**

Twenty-one original studies (four in early psychosis) were included in the review. We report on types of traumas addressed; treatments provided and key outcomes; participant perspectives on treatments and contextual factors impacting implementation such as characteristics of providers who delivered treatments and cultural considerations.

**Conclusions:**

Identified studies suggest a growing evidence base for addressing PTSD symptoms in psychosis; however, treatments are lengthy and have complex training requirements which pose challenges for implementation in routine care, particularly in time-limited early psychosis programs. More work is needed regarding identifying optimal treatment dose and understanding which treatment elements contribute to outcomes and are tolerable for participants.

## Introduction

Individuals living with psychosis are three times as likely to report a history of childhood trauma than the general population,^1,2^ and rates of posttraumatic stress disorder (PTSD) are as high as 29% (compared to ~8% in the general population).^3,4^ Importantly, trauma may contribute to the onset and content of psychosis-related experiences (eg, hallucinations reflecting trauma-related themes, such as hearing the voice of a perpetrator).^5^ Additionally, individuals may experience unique psychosis-related traumas, such as forced restraint in hospital settings.^6–9^ Despite this, individuals with psychosis have often been excluded from clinical trials examining efficacy of trauma-focused treatments.^10^ For example, in a randomized controlled trial (RCT) of psychotherapies for PTSD, a large number of individuals with psychosis self-presented to the trial (despite being ultimately excluded) and viewed trauma as an important part of their experience that had not been adequately addressed as part of their care.^11^ Studies also suggest lower screening rates and lack of incorporation of trauma history into treatment planning when psychosis is present.^12^ This lack of detection may reflect conceptualizations that separate psychosis from trauma-related consequences, despite evidence suggesting that traumatic experiences can shape the onset and content of psychotic symptoms.^5,13,14^ Recent studies have begun to address this gap by examining safety and efficacy of trauma treatments in individuals with lifetime diagnosis of psychosis.^15,16^

While some narrative and systematic reviews^16–19^ have begun to synthesize these findings, they have primarily focused on chronic or later-stage psychosis and have not adequately addressed how these interventions function in the context of early psychosis (individuals within 5 years of illness onset or who are enrolled in early intervention services (EIS) for recent onset psychosis^20^), a clinically and developmentally distinct period. Additionally, while these reviews have considered symptomatic outcomes (either psychosis symptoms alone^18^ or PTSD symptoms and other outcomes such as anxiety, depression^19^), they have not incorporated participant perspectives or contextual factors (eg, provider characteristics, cultural considerations) that are critical to understanding feasibility and acceptability of trauma-focused treatments. This represents a critical gap in the literature.

Early psychosis, typically starting in adolescence or young adulthood, remains largely unexplored with respect to trauma treatment.^20^ The developmental context presents varying needs for treatment due to proximity to the first episode, varying levels of engagement, possible family involvement in treatment, and life changes associated with the developmental period (eg, starting college or work).^21^ The wide age range of individuals with early psychosis complicates the application of trauma treatments that are often developed separately for adult and child/adolescent populations. Additionally, EIS, which provide coordinated specialty care, the leading team-based model of care for individuals with early psychosis, often operate under time-limited frameworks, typically 2–5 years, which may favor briefer, scalable trauma interventions over complex protocols.^22,23^ Providers in these settings frequently express uncertainty in how to address trauma, further underscoring the need for a better understanding of the current evidence base in this area.^24^

While exposure to trauma has been consistently linked to the development and severity of psychosis, including through dose–response relationships and trauma-related content in psychosis symptoms,^1,2,5^ this review focuses specifically on trauma-focused treatments designed to target comorbid PTSD or subclinical posttraumatic stress (PTS) symptoms (eg, re-experiencing, avoidance, hyper-arousal) in individuals diagnosed with schizophrenia spectrum disorders or affective disorders with psychosis. We acknowledge that psychotic symptoms themselves may be trauma-related in origin or expression; however, our aim was to examine whether existing interventions explicitly designed to address PTSD symptoms have been applied across stages of psychosis, particularly in early intervention contexts.

We pay particular attention to comparisons between early and later stages of illness, defining early psychosis as individuals who are within 5 years of illness onset or who are enrolled in EIS for recent onset psychosis. We report on types of traumas targeted; treatment provided and key outcomes; participant perspectives on trauma-focused treatments; and contextual factors impacting implementation such as characteristics of providers who delivered the treatments and cultural considerations. Given the limited number and quality of available studies, a scoping review methodology was selected to capture the breadth of the literature, highlight implementation trends, and identify key gaps and priorities for future research and practice.^25^

## Methods

A review protocol was preregistered in Open Science Framework (https://doi.org/10.17605/OSF.IO/6CUX5). Review questions set in advance focused on types of traumas addressed and determination of need for treatment, and treatments provided and key outcomes. We additionally included information regarding participant perspectives on treatments offered and considerations of contextual factors impacting implementation, such as characteristics of providers involved in delivering the treatments and cultural considerations. Our search followed the PRISMA-SRc guidelines for scoping reviews.^26^

### Eligibility Criteria

Included studies were limited to peer-reviewed, original studies that examined outcomes of psychotherapies targeting PTSD or subclinical PTSD symptoms (PTS) in individuals diagnosed with schizophrenia spectrum disorders or affective disorders with psychosis. Studies had to be written in English and published between 1980 and 2025. Randomized, uncontrolled studies, and case series were included. Qualitative studies were considered if they included participant perspectives regarding a specific trauma focused therapy they had received. We excluded dissertations, reviews, single case studies; secondary data analyses of primary studies; and studies considering interventions that are not psychotherapy (eg, medication trials).

### Study Search and Selection

Three databases (PubMed, PsycINFO, Web of Science) were searched in December 2024 using specific keywords related to psychosis, trauma, and treatment: (Psychosis OR Psychoses OR Psychotic OR Schizophren^∗^) AND (trauma^∗^ OR PTSD OR “Posttraumatic stress” OR “Post traumatic stress”) AND (treatment OR intervention OR therapy). The full text records and reference lists were examined for additional papers. Searchers were re-run in June 2025 to capture any newly published studies. Two reviewers (SJ and RB) independently screened all titles and abstracts. Discrepancies were resolved through discussion. SJ and RB independently reviewed full-text articles and used a data extraction template which they jointly developed on Covidence (a review software) to record relevant study information for selected articles. We extracted data on the year of publication, study location, study sample, treatment used, key outcomes, participant perspectives on the treatment, and contextual considerations (provider characteristics, cultural factors).

### Quality Assessment

Study quality was assessed using the Mixed Methods Appraisal Tool (MMAT),^27^ which is designed to assess quantitative, qualitative, and mixed-method studies using a single tool. Studies were scored based on four assessment criteria (see Supplement) by two raters (SJ and BT). Discrepancies were resolved through discussion. A summary score was calculated by dividing number of criteria met (score of “yes”) by five to produce a range of scores (0, 20, 40, 60, 80 and 100%).

## Results

### Overview

**Figure 1.**
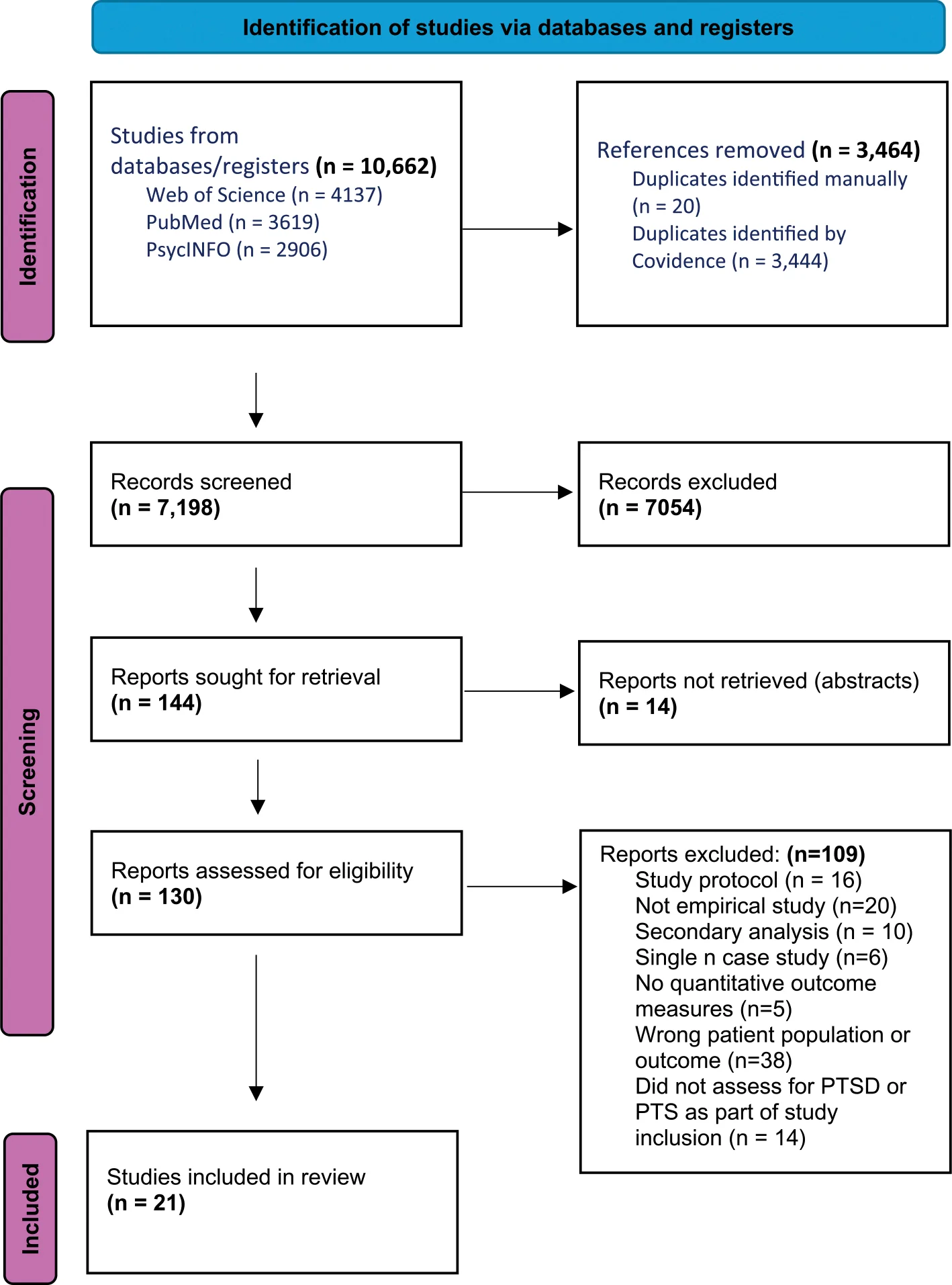
Preferred Reporting Items for Systematic Reviews and Meta-Analyses Flowchart

We identified 21 original studies for inclusion in the review (see Figure 1 for PRISMA flowchart). Table 1 includes a summary of study characteristics. Of these, only four were conducted in individuals with early psychosis.^28,29,43,44^ Two included participants with comorbid PTSD^28,29^ and two with subclinical PTSD symptoms (PTS).^43,44^ Only one was a randomized controlled trial.^43^ Notably two studies that tested trauma-focused treatments (a qualitative study on Narrative Exposure Therapy^48^ and a quantitative study onadaptedProlongedExposure^42^) in early psychosis were excluded due to having inclusion criteria of only having experienced a lifetime traumatic event,^42,48^ without assessing PTSD or other trauma-related symptoms prior to offering the intervention.

Most studies (n=17) were conducted in individuals with a lifetime diagnosis of psychosis who either had comorbid PTSD determined by a formal assessment or identified by their primary therapist without a specified assessment tool (n=13) or experienced significant PTS symptoms (n=4, of these, one had originally required a full diagnosis but adjusted the criteria to subthreshold symptoms after difficulties with recruitment^48^). Most were single group pre-post study designs (n=8) and only five were RCTs. The majority were conducted in outpatient settings, with only one in forensic services,^42^ one on the inpatient unit,^37^ and two in assertive community treatment teams.^34,40^ Most treatments used an individual therapy approach, with only two group-based interventions (eg, a non-randomized controlled trial testing a non-exposure-based Skills Training in Affective and Interpersonal Regulation (STAIR) intervention on the inpatient unit^37^; and a pre-post study testing a mixed group and individual exposure-based cognitive behavioral therapy (CBT) intervention for PTSD^49^).

### Types of Traumas Addressed

The majority of studies addressed childhood or adulthood traumas unrelated to psychosis (n=19), while two did not specify the traumas addressed.^39,44^ Most index traumas were interpersonal in nature, including sexual or physical abuse. Other trauma types included combat or war related experiences, motor vehicle accidents, and interactions with the criminal justice system. Of the19, six studies (including three studies in early psychosis^43,28,29^) also considered psychosis related traumas, such as physical seclusion or restraint in a psychiatric hospital and experience of a distressing psychotic episode as an index trauma.^36,40,48^ In one study conducted in a forensic setting, the index offense (eg, committing a crime in the context of worsening psychosis symptoms) was considered to be a potentially traumatic event warranting treatment.^42^

### Treatments Provided and Key Outcomes

Broadly, treatments included (1) those specified by National Institute for Health and Care Excellence (NICE) guidelines for treating trauma-related symptoms in psychosis, (2) integrative approaches targeting trauma and psychosis symptoms in the same protocol, and (3) experimental approaches. NICE PTSD guidelines specify the use of TF-CBT (including prolonged exposure [PE], narrative exposure therapy [NET], cognitive processing therapy [CPT], and cognitive therapy [CT]) and eye movement and desensitization reprocessing (EMDR).^50^ Below we provide an overview of the treatments separated for early psychosis and those with a lifetime diagnosis.

### Early Psychosis

Treatments in early psychosis consisted of integrative treatments, treatments that target psychosis and trauma-related symptoms in the same protocol (Trauma-integrated cognitive behavioral therapy for psychosis [TI-CBTp^44^] and adapted Eye movement and desensitization reprocessing for psychosis [EMDRp^43^]) or experimental approaches (Acceptance and commitment therapy [ACT^31^], Trauma-integrated psychotherapy for psychosis [TRIPP^28^]). Table 2 includes a description of the treatments.

#### Eye Movement and Desensitization Reprocessing (EMDRp).

In the only RCT conducted in early psychosis comparing EMDRp+TAU to TAU within the context of an early intervention program (EIS) in individuals with PTS, there were signals of promise of efficacy of EMDR on PTS symptoms, psychotic symptoms, and subjective recovery at 6 months, which continued at 12 months but were less pronounced.^43^ Dropout rates were 27% due to general engagement difficulties with EIS and wanting to withdraw from the trial or prioritize other therapies offered.

#### Trauma-Integrated Cognitive Behavioral Therapy for Psychosis (TI-CBTp).

In another pre-post study conducted within an EIS program in individuals with PTS, TI-CBTp (a treatment combining CBT for psychosis [CBTp] and TF-CBT, a youth focused trauma treatment) showed trends towards improvements in PTS symptoms for individuals who began the trauma-focused aspect of the intervention (although data regarding PTS symptoms was limited due to changes in the measure over time). It was also unclear which component of the intervention (eg, involvement of a support person or exposure) or number of sessions participants received.^44^ Dropout rates were not specified.

#### Trauma-Integrated Psychotherapy for Psychosis (TRIPP).

A mixed-methods study reported on pre-post findings from individuals with comorbid PTSD receiving TRIPP and found clinically significant improvements in PTSD and psychosis symptoms for 6 of 8 participants (one did not want to complete assessments).^28^ All participants reported feeling distressed during session and half reported feeling distressed or having temporary exacerbation of symptoms outside of session. Despite this, they perceived the therapy as worthwhile and noted they would recommend it to others.^28^

#### Acceptance and Commitment Therapy (ACT).

Another case series examining the impact of ACT in three individuals with early psychosis and PTSD found reliable improvements in PTSD symptoms; however the study did not provide information on the nature of psychosis symptoms of the participants and the symptom presentation of two of the participants appeared more consistent with severe PTSD.^29^

### Lifetime Diagnosis

Treatments in individuals with a lifetime diagnosis of psychosis included treatments recommended by NICE guidelines, as mentioned above (ie, PE, EMDR, NET, CPT), Skills Training in Affective and Interpersonal Regulation (STAIR), variations of a CBT protocol for PTSD, integrative treatments (Trauma-focused cognitive behavioral therapy for psychosis [Tf-CBTp]), and experimental approaches (imagery rescripting [ImRs]). For treatments involving a memory processing component, transient increases in symptoms were common,^43,46,28^ but only one study involving a 3-session imagery rescripting protocol (ImRs) reported a serious adverse event that was study related.^47^

#### Prolonged Exposure and Eye Movement and Desensitization Reprocessing (PE and EMDR).

Five studies tested PE and/or EMDR, and were conducted according to standard treatment protocols without a stabilization component prior to implementing the intervention.^33,36,38,40,42^ Number of sessions ranged from 8 to 15, with one study allowing up to 15 sessions if participants continued to report significant PTS symptoms around the eighth session, but only one participant needed this.^33^ Across studies, participants showed general improvements in PTSD symptoms, and of those that measured psychosis symptoms, two reported no changes^38,42^ and one EMDR pre-post study reported small, significant reductions in auditory verbal hallucinations and delusions, but not paranoid ideation at post-treatment.^36^ When compared directly in an RCT, individuals receiving PE and EMDR both showed improvements in PTSD symptoms at posttreatment and 6-month follow-up, but those in the PE group were more likely to achieve full remission of PTSD.^40^ Across studies, dropout rates ranged from 18% to 35%, with common reasons including not believing rationale for the treatment, symptoms not improving or improving after a few sessions, and difficulties with session attendance.^33,36^

#### Narrative Exposure Therapy (NET).

In one pre-post study testing NET, participants experienced decreases in PTSD symptom severity at 1-month post-treatment and 7-month follow-up, and 48% were in remission at 7-months.^34^ Only two dropped out. However, one major limitation of this study is that most participants (65%) had major depressive disorder.

#### Cognitive Processing Therapy with Written Narrative Component (CPT+A).

In a pre-post study testing CPT+A, participants experienced significant decreases in PTSD symptoms in intent-to-treat analyses, and psychosis symptoms were not measured. Dropout rates were 33%, and most (56%) had major depression with the remaining having bipolar disorder. Notably, there are two versions of CPT, one including a written narrative component (CPT+A) and one without (CPT-C). A recent meta-analysis comparing the two protocols, reported no significant difference in treatment effect sizes or dropout rates.^52^

#### Skills Training in Affective and Interpersonal Regulation (STAIR).

Another non-randomized control trial compared STAIR in a group format on the inpatient unit to a matched control group who received supportive therapy, found significant improvements in PTS and positive psychosis symptoms in the STAIR group, but not the control.^37^ They did not report dropouts, did not account for confounders in analysis or provide demographic data regarding baseline differences between groups, and did not specify how PTSD diagnosis was ascertained.^37^

#### Cognitive Behavioral Therapy for PTSD (CBT for PTSD).

Six studies tested variations of a CBT for PTSD protocol, that included cognitive restructuring (CBT-P) with or without a video to facilitate psychoeducation^31,39,41,48,30^ or had an exposure-based component preceded by stabilizing interventions.^49^ Although 77% of treatment completers in the exposure-based intervention no longer met criteria for PTSD or had clinically significant reductions in PTSD symptoms, there was a 35% dropout rate and participants that were part of a day treatment program were more likely to complete the intensive treatment protocol that consisted of group and individual sessions than individuals enrolled in outpatient clinics.^32^

In pre-post studies^31,30^ and two RCTs comparing CBT-P to treatment as usual (TAU)^41^ and a brief three session psychoeducation and breathing intervention (BRF),^39^ respectively, participants receiving CBT-P reported improvements in PTSD symptoms at post, 3, and 6 months and decreases in negative trauma related cognitions, and CBT-P was significantly more effective than BRF. Psychosis symptoms were not measured. Dropout rates ranged from 14% to 26% and the samples were predominantly comprised of individuals with major mood disorders (41%–85%) and comorbid PTSD. In an RCT testing CBT-P compared to TAU involving participants with only schizophrenia or schizoaffective disorder and comorbid PTSD (which was later adjusted to PTS due to difficulties with recruitment), there was no significant difference between groups on PTSD and psychosis symptoms (both experienced improvements in symptoms).^48^ Post-hoc analyses conducted on a subsample with PTSD also did not yield significant effects. The authors posited that emotional processing of traumatic memories may be an important treatment component and that it may be important to assess stability of PTS symptoms over a period of time prior to determining eligibility.^48^

#### Trauma-Focused Cognitive Behavioral Therapy for Psychosis (Tf-CBTp).

Tf-CBTp, which combines cognitive behavioral therapy for psychosis (CBTp) and trauma-focused CBT (TF-CBT; consisting of exposure, imagery, or cognitive techniques), was initially tested in a case series and showed improvements in PTS symptoms from severe to moderate-to-severe at post and 9 months.^45^ Approximately half showed reliable improvements in delusional beliefs and 25% in voice severity at post-treatment and 9 months.^45^ This approach was tested as part of regular services in the UK in a pre-post study, and 69% of the sample experienced clinically significant changes in symptoms and 38% no longer met criteria for PTSD.^46^ Symptoms worsened for three individuals due to recurrence of trauma. Psychosis symptoms were not measured and there were no dropouts.^46^ This protocol is currently being tested in a multisite RCT, including in individuals with early psychosis.^53^

#### Imagery Rescripting (ImRs).

Lastly, a multiple baseline study of ImRs found clinically significant change in PTS symptoms in 58% of participants, 3 out of 12 no longer met criteria for PTSD, and there were no dropouts.^47^ The authors note that despite promising results, the protocol maybe better suited when integrated into another therapy rather than as a standalone and participants may benefit from distress tolerance skills prior to receiving ImRs.^47^

### Contextual Considerations

#### Participant Perspectives.

Seven studies reported information regarding participant perspectives on the treatments, including protocols targeting trauma and psychosis symptoms simultaneously,^45,46^ exposure-based CBT interventions,^32,33^ EMDR,^42,54^ imagery rescripting,^47^ and TRIPP.^28^ Only one was in early psychosis.^28^ None of the interventions were co-designed with participants. Studies testing exposure-based interventions included perspectives of treatment completers and non-completers^32^ and those whose symptoms improved or did not.^33^ In a study examining perspectives of treatment completers of an exposure-based CBT intervention who experienced improvement in symptoms versus those who did not, both groups reported benefits over time, including decreases in overall distress and being better able to speak about the trauma.^33^ Similarly, broadly across all treatments, participants noted temporary increases in distress, but reported that the treatment was helpful and that they would recommend it to others.

Specifically for EMDR, justice involved participants valued the privacy offered by this treatment in a forensic environment where this was not commonplace.^54^ Regarding a brief standalone 3-session imagery rescripting protocol, participants rated the treatment as acceptable, but some struggled with the rationale which asks participants to change aspects of an image from a traumaticevent.^47^ They reported that this change signified to them a denial that the trauma occurred. The authors suggested that the protocol may be more effective if integrated into a longer term treatment to allow time for understanding treatment rationale.^47^ Strategies suggested by participants to increase the tolerability of treatments included having breaks during session, incorporating external supports before and after the session, gradual use of different techniques, and emphasizing the value of incremental change.^46^ Two studies also highlighted the importance of the therapeutic relationship.^33,46^

### Characteristics of Clinicians Who Delivered the Treatment

Three studies did not report provider characteristics,^33,37,28^ and 11 did not report any fidelity checks on therapist implementation of the treatment.^33–37,44–47,28,29^ Of the studies that reported provider characteristics, five included the first author or members of the study team,^35,38,41,45,47,29^ five specified that frontline clinicians (testing CBT-P and NET)^31,34,39^ or clinical psychology trainees delivered the therapy as part of usual care (TI-CBTp and Tf-CBTp),^44,46^ and eight described providers of various levels of experience with psychosis or trauma-focused treatments but did not specify whether they were frontline clinicians or study therapists (CBT-P, CBT for PTSD with exposure, PE, EMDR, EMDRp).^32,36,40–43,48,30^

For studies that reported training of frontline clinicians, training of CBT-P included a 2 day training and weekly group supervision^39^ and NET included a 3 day training and 10, 90-min group supervision sessions.^34^ In a trial that did not specify whether providers were study therapists or primary clinicians, clinical psychologists and a psychiatrist who were not previously trained in PE or EMDR received a four day training and treated at least 2 supervised cases per intervention prior to delivering both treatments during the trial.^40^ Rosenberg et al.^30^ also emphasized that buy-in and education about trauma for the rest of the clinical team that is not directly involved in delivering the intervention is essential in order to help generalize skill use.

### Cultural Considerations

Four studies highlighted important cultural considerations. In one study, two participants noted preference for working with therapists having similar lived experience, particularly if their trauma is due to homophobia or racism.^46^ One also highlighted alienation from their own culture clients may experience following a trauma and the need to discuss this in therapy.^46^ Another study examined feasibility and acceptability of delivering a CBT intervention for PTSD with Black participants.^31^ Although the manual was not changed, the intervention was delivered by therapists who identified as Black, and sociocultural factors were regularly discussed in supervision (eg, “cultural paranoia” and mistrust of the healthcare system).^31^ “Cultural paranoia” refers to the fact that normative levels of paranoia (ie, suspiciousness and mistrust of others regardless of mental health status) may be higher in Black individuals as an adaptive response to racial oppression.^55,56^ Another qualitative paper mentioned the importance of exploring acceptability of the therapy in future research for different cultural groups, such as the Maori population in New Zealand, given connections between colonization, trauma, and mental health.^54^ Lastly, one PE study noted higher dropout rates among younger and OEF/OIF (Operation Enduring Freedom/Operation Iraqi Freedom) veterans, but did not have any data on why this was the case.^33^

## Discussion

Our review identified four studies (one RCT) in early psychosis and seventeen studies (five RCTs) in individuals with a lifetime diagnosis of psychosis. Fifteen included individuals with comorbid PTSD and six with subclinical PTSD symptoms (PTS). Given the limited number of studies and methodological variability (eg, few RCTs and lack of comparison groups, small sample sizes, study heterogeneity, limited reporting on treatment fidelity), findings should be interpreted as preliminary rather than conclusive. These limitations reflect broader challenges in studying trauma-related symptoms in psychosis, particularly in team-based early intervention contexts where randomization at the individual participant level may not always be feasible and highlight the need for pragmatic trial designs that can accommodate these constraints.

Despite these limitations, this review highlights recent advances made in the psychosis trauma literature. Promising emerging treatments in early psychosis include integrative approaches such as TI-CBTp and EMDRp that target psychosis and trauma symptoms simultaneously or sequentially as part of standard care for early psychosis.^43,44^ In particular, TI-CBTp has a youth focus that can be helpful when working with adolescents and young adults with early psychosis and has the option of including a support person as part of the treatment (although it was not specified in the study how many participants included a support person as part of their treatment or which components of the treatment they had received).^44^ A recent systematic review examining the inclusion of social support persons as part of PTSD treatment in adults identified only eight studies and found that active involvement of a significant other in treatment was associated with reduced PTSD symptoms.^57^ More research is needed to understand the inclusion of social support persons in treatment and impacts on treatment efficacy, particularly in youth.

Research conducted with individuals with a lifetime diagnosis suggests that traditional evidence-based treatments for PTSD (eg, PE or EMDR) can be safely implemented with little or no adaptations. However, as in studies with the general population, these treatments are associated with high drop outrates (18%–35% in psychosis and ~20% in the general population with the highest rates for CPT at 34% and PE at 29% and lowest rates for NET at 7% in the general population) and may not be appropriate for everyone.^58^ This suggests that while these treatments may be safe, they are not universally tolerable, particularly in populations with complex needs.

Methodological limitations across studies limit interpretability. Common issues include lack of comparison groups, small sample sizes, and limited reporting of treatment fidelity. Additional considerations include the heterogeneity of diagnostic characteristics in the sample of psychosis specific studies, including studies where the majority of individuals have depression^31,34,35,30^ and varying inclusion criteria where some studies only include individuals with comorbid PTSD, while others include PTS and some only require the presence of a traumatic event that the individual reports as distressing.^59^ These inconsistencies complicate cross-study comparisons and highlight the need for more rigorous, standardized trials.

Recent studies have called for the need for personalized care to address trauma-related symptoms, rather than a one-size fits all approach and to consider which components of interventions may be most effective for certain individuals (eg, focusing on fear pathways or memory disturbances in PTSD versus identity disturbances in complex PTSD).^60,61^ Emerging treatments with promising findings addressing complex PTSD include Enhanced Skills Training in Affective and Interpersonal Regulation (ESTAIR),^62^ which is currently being tested in Scotland in individuals with complex PTSD and psychosis (NCT05281640). There is also a question of whether exposure is necessary and whether non-exposure based treatments such as Present-Centered Therapy (PCT), Interpersonal Psychotherapy (IPT), Acceptance and Commitment Therapy (ACT), or equine assisted therapy^63^ may be potential alternatives.^64^ Of these, only ACT has been tested in a case series with individuals with early psychosis and showed promising preliminary results in improving PTSD symptoms.^29,65^ Notably, a CBT based intervention without an exposure element (CBT-P) was found to be no different from treatment as usual, which may suggest that an emotional processing component may be necessary.^48^

Additionally, although several treatments have been shown to be effective for PTSD, there are several barriers to implementation including complexity of protocols, training requirements and deciding who can deliver these treatments, identifying which individuals are most likely to benefit, and deciding when to provide these treatments. Of the studies identified, only five explicitly stated that they included frontline clinicians as treatment providers, raising questions about scalability and sustainability in routine care.^31,34,39,44,46^ These considerations are especially important when working with individuals with early psychosis, for whom the evidence base is much smaller. Existing studies in early psychosis have included integrative treatments that combine interventions for psychosis with interventions for trauma-related symptoms,^44,53^ adding psychosis-specific elements and stabilizing interventions to treatment protocols,^51^ or using experimental approaches that target experiential avoidance^29^ and pursuit of values or involve creation of a timeline of major life events.^28^ Although integrative protocols have the potential to address psychosis and trauma-related symptoms simultaneously, they are quite complex and lengthy, which is a major barrier to implementation, specifically in time limited early intervention programs where staff turnaround is common.^44^ Similarly, treatments like EMDR require a long, expensive training and certification process that may not be feasible in the public sector. There is a need to identify which treatment protocols are most feasible to implement and when.

To identify appropriate treatments and attempt to address high dropout rates, consideration of participant perspectives and cultural factors is essential. Only seven studies considered participant perspectives, which included reports of temporary distress but overall positive views of treatment. EMDR was viewed as particularly impactful in a forensic setting due to the privacy offered by the treatment.^42,54^ Four studies highlighted important cultural considerations, such as therapist-client concordance particularly if participants had experienced trauma related to homophobia or racism,^46^ recognition of normative “cultural paranoia” in Black participants,^31^ and differences in treatment retention based on age.^33^ More studies are needed to understand how different cultural groups respond to treatment and what modifications may be necessary.

Some adaptations to PTSD treatments identified in a recent systematic review in the general population included using culturally relevant metaphors and adapting language, addressing stigma, awareness of spirituality and its impact on trauma responses, offering services in settings outside of traditional services such as faith-based organizations, and providing psychoeducation around trauma that draws on sociocultural history.^66^ Additionally, specifically for individuals with early psychosis, youth-specific adaptations may be especially important to address fluctuating motivation and engagement difficulties, developmental transitions, such as career or school choice and intimate relationships, and involvement of support persons in the therapy.^67^ Additionally, future research should move beyond categorical distinctions between PTSD and psychosis to develop integrative models that account for experiences of individuals whose trauma and psychosis related symptoms are intertwined.

### Limitations

Limitations of the current review include our conceptualization of early psychosis papers as those where most of the sample was referred to as with early psychosis or enrolled in EIS. Some studies we characterized as lifetime may have included individuals with early psychosis; however, since results were not reported separately for these individuals, we considered them to be more representative of individuals with a lifetime diagnosis. We also excluded studies where inclusion criteria did not include screening for PTS symptoms, which caused us to exclude potential studies addressing trauma-related experiences in early psychosis.^59,68–70^ Additionally, we did not consider studies conducted in individuals with clinical high risk (CHR); however, to our knowledge Folk et al^44^ included a small sample of individuals with CHR and only two other studies testing EMDR have been conducted in CHR.^71,72^ Dialogical approaches were excluded from this review due to their distinct conceptual focus on relational dynamics with voices (that may or may not be trauma-related) rather than targeting PTSD symptoms directly.^73–75^ Lastly, the included studies varied significantly in design and sample characteristics, limiting the strength of conclusions.

## Conclusions

Our scoping review suggests that there is a growing evidence base for addressing PTSD symptoms in psychosis; however, the current evidence base remains limited in scope and quality, and findings should be interpreted as preliminary. More work is needed to identify appropriate treatments that can be implemented into routine care and will be tolerable to participants, particularly in individuals with early psychosis. Additionally special attention must be paid to sample characteristics across studies, including diagnostic heterogeneity and study inclusion criteria, when interpreting efficacy of various treatment approaches. Lastly, it is essential to understand how cultural factors impact tolerability of certain treatments and how to enhance existing interventions with consideration of these factors. By explicitly comparing findings across illness stages and integrating contextual factors such as culture and participant perspectives, this review provides a foundation for future research in trauma-focused treatment for psychosis.

## Supplementary Material

supplement

Supplementary material is available at https://academic.oup.com/schizophreniabulletin.

## Figures and Tables

**Table 1.** Characteristics of Selected Studies

Study	Study Type	Country	N	Trauma Treatment	Number of sessions	Participant diagnoses (primary)	PTSD Outcomes	Psychosis Outcomes	Other Outcomes	Dropout rates
**Participants had Comorbid Posttraumatic Stress Disorder (PTSD)**										
***Early Psychosis***										
Tong 2017^28^	PP	AUS	8	TRIPP	Ranged from 18 to 24 months	Schizophrenia (25%), schizoaffective disorder (38%), bipolar with psychotic features (12.5%), psychotic disorder NOS (12.5%)	6 participants had clinically significant improvements on the CAPS total severity score (15 point change), 1 did not improve, and 1 did not complete measures	4 participants minimally improved on the BPRS (symptoms reduced 25% from baseline), 2 much improved (reduction of 50%-55%), 1 did not improve, and 1 did not have complete measures	n/a	Sample was limited to treatment completers
Jansen 2017^29^	CS	ND	3	ACT	12	Schizophrenia (n = 1); Unspecified psychosis and mixed personality disorder (n = 1); Paranoid schizophrenia (n = 1)	Reliable improvements were found for PTSD symptoms (measured by IES-R).	Not measured	Reliable improvements were found for anxiety and depression symptoms.	No dropouts
***Lifetime Diagnosis***										
Rosenberg 2004^30^	PP	US	22	CBT-P	12–16	Major depression (41%), Schizophrenia (23%), Schizoaffective (18%), Bipolar disorder (14%), Psychotic disorder NOS (4%)	Significant reductions in CAPS based PTSD diagnosis from 100% at baseline to 64% at post and 50% at 3 month follow-up.	Not measured	Significant reductions in depression and anxiety from baseline to 3 months follow-up, but not baseline to post.	Treatment retention was 86% (completed at least 6 sessions). Dropouts due to treatment related distress or unrelated stressors (child care, disruptions in living situation).
Lu 2009^31^	PP	US	14	CBT-P	12–16	Bipolar or major depressive disorder (79%), Schizophrenia or schizoaffective disorder (21%)	77% of participants improved on PCL scores from baseline to post-treatment (7.8 points), and 83% improved at 3 (14.6 points) and 6 months (10.4 points) posttreatment. The proportion of the sample meeting PTSD diagnostic criteria using PCL scores changed from 100% at baseline to 69% at posttreatment, 33% at 3-month post-treatment and 58% at the 6-month follow-up.	Not measured	62% of the participants had less severe depression at post-treatment, 75% at 3 months post-treatment, and 92% at 6 months	5 of 19 (26%) participants dropped out (not completing at least 6 sessions) due to leaving the program, paralysis, or ongoing litigation as a result of the trauma.
Frueh 2009^32^	PP	US	20	CBT for PTSD (with exposure)	22 group and individual over 11 weeks	Rates not specified (all had PTSD and Schizophrenia or Schizoaffective disorder)	Significant PTSD symptom improvement, maintained at 3-month follow-up. 10 of 13 completers no longer met criteria for PTSD or were considered treatment responders (at least 15 pt decrease in CAPS).	Not measured	No significant improvements on depression or general anxiety	Treatment completion was 65% (completed at least 70% of sessions). None dropped out during exposure therapy. Those in day treatment were more likely to complete than in outpatient.
Grubaugh 2017^33^	PP	US	14	PE	10–15	Rates not specified (All had diagnosis of psychotic disorder and PTSD)	Significant pre- to post-changes in CAPS severity and PCL scores in treatment completers (n = 9).	Not measured	n/a	6 participants did not complete treatment (at least 8 sessions) due to transportation problems, being lost to follow-up or wanting to withdraw, experiencing symptom improvement. Younger and OEF/OIF veterans and those with higher levels of PTSD symptoms were more likely to drop out. Most dropped out prior to beginning imaginal exposure.
Mauritz 2021^34^	PP	ND	23	NET	16	Major depressive disorder (65%), Schizophrenia spectrum disorder (17%), Bipolar disorder (17%)	Mean PTSD severity decreased from 37.9 at baseline to 31.9 at 1 month post-treatment, and 24.5 at 7 month follow-up. Eleven participants were in remission for PTSD at 7 months.	SMI symptoms (measured with the HoNOS) decreased between baseline and 7 month follow-up.	5 of 11 participants who were in remission for PTSD at 7 months, were also in remission for major depression. Global functioning significantly increased. Suicide risk shifted from high and medium risk to low risk. Substance misuse decreased from 30% to 24%.	Of 23 patients, 2 dropped out after 1 and 4 sessions.
Nishith 2024^35^	PP	US	49	CPT + A (with exposure)	12	Major depressive disorder (56%, of 39 who started CPT), Bipolar disorder with psychotic features (44%)	Results showed statistically significant changes in PTSD symptoms in intent to treat analyses	Not measured	Depressive symptoms and general psychiatric functioning improved in intent to treat analyses.	Of the 39 participants who started CPT, 33% dropped out.
vandenBerg 2012^36^	PP	ND	27	EMDR	6	(Schizophrenia 22%, Schizoaffective 22%, Delusional disorder 3.7%, Psychotic disorder NOS 52%) and Comorbid PTSD	Only 5 of 22 completers (22.7%) still met criteria for PTSD at post. CAPS PTSD score was reduced (42.4% in intention to treat analysis and 52.6% in completers).	There were small, significant reductions in auditory verbal hallucinations and delusions, but no effects on paranoid ideation	Depression and anxiety improved.	The dropout rate was 18.5%. Reasons included not believing rationale for treatment, symptoms not improving, traveling, symptoms improving after 2 sessions, lack of session attendance.
Trappier 2007^37^	NRC	US	24	STAIR vs 12 weeks of supportive therapy	12 group	Rates not specified	There were significant improvements on PTS symptoms on the IES in the CBT group and on the intrusion and avoidance subscales, but not in the supportive psychotherapy group.	There was a significant decrease in positive symptoms in the CBT group, but not in the supportive psychotherapy group.	Both groups improved on somatic concern, anxiety, hostility, suspiciousness, and uncooperativeness. Those receiving STAIR also improved on emotional withdrawal, tension, depressive mood and unusual thought content.	Not reported
deBont 2013^38^	WGC	ND	10	PE vs EMDR	12	Schizophrenia (40%), Psychosis NOS (40%), Schizoaffective (10%), Bipolar disorder (10%)	Intent to treat analysis showed that both treatments significantly reduced PTSD symptom severity, which was sustained at 3 months. Of 8 completers, 3 in PE and 3 in EMDR no longer met PTSD criteria.	No change in psychotic symptoms (fairly low at baseline).	Decrease in general psychopathology.	80% of participants completed the full intervention
Mueser 2015^39^	RCT	US	201	CBT-P (12–16 sessions) vs BRF (3 sessions); no control	CBT-*p* = 12–16, BRF = 3	Major Mood Disorder (49%), Schizophrenia spectrum disorder (28%), Major mood and Borderline personality disorder (21%), Schizophrenia spectrum and Borderline personality disorder (7.3%)	Those who received CBT-P showed significantly greater reductions in PTSD symptoms than those who received BRF, although both groups showed improvements. They also had greater remission rates and improvement in knowledge of PTSD.	Not measured	CBT-P group had more improvements on overall functioning (GAF) and social functioning (CAPS). Both groups improved over time on depression symptoms from post to follow-up.	4 dropped out of BRF (only received 1 session) and 22 dropped out of CBT-P (received 1–5 sessions)
van den Berg 2015^40^	RCT	ND	155	PE vs EMDR vs waitlist control	8	Schizophrenia (61.3%) or Schizoaffective disorder (29%)	There were significant improvements on mean CAPS total scores for both treatments compared with waitlist control at post-treatment and 6-month follow-up. Participants in PE, but not those in EMDR, were more likely to achieve full remission of PTSD than participants in the waitlist condition.	Not measured	n/a	There was no difference in dropouts between the PE (n = 13, 24%) and EMDR (n = 11, 20%).
Mueser 2008^41^	RCT	US	108	CBT-P vs TAU	12–16	Major mood disorder (85%) or Schizophreni-a/Schizoaffective disorder (15%) and PTSD	CBT-P was no more effective than TAU at eliminating PTSD diagnosis, but was significantly better at reducing PTSD symptoms and negative trauma related cognitions and improving knowledge of PTSD. The effects of CBT-P on PTSD were strongest in clients with severe PTSD.	Not measured	CBT was more effective than TAU at reducing depression and anxiety	81% of participants assigned to CBT-P group participated. There was a dropout rate of 19%.
Every-Palmer 2024^42^	RCT	NZ	24	EMDR vs waitlist control	9	Schizophrenia (58%), Bipolar disorder with psychotic features (17%), Unspecified psychosis (13%), Schizoaffective disorder (8%)	CAPS PTSD Scale mean scores were lower in the EMDR group compared with the control group after 6 months.	No significant differences between groups on psychotic symptoms, although trends favored EMDR	There was some evidence of reduced depressive symptoms (BDI-II) in the EMDR group after 10 weeks, but differences were not statistically significant after 6 months.	No dropouts
**Participants had Significant Posttraumatic Stress (PTS) Symptoms**										
***Early Psychosis***										
Varese 2024^43^	RCT	UK	60	EMDRp vs TAU	16	rates not specified	There were signals of promise of efficacy of EMDRp vs TAU on PTSD symptoms at 6 months, which were less pronounced but remained at 12 months. EMDRp was associated with lower odds of meeting criteria for PTSD on the ITQ and PCL-5 at both 6-months and 12-months and with lower odds of meeting criteria for CPTSD at the 12-months.	There were signals of promise of efficacy of EMDRp vs TAU for total psychotic symptoms and subjective recovery from psychosis at 6 months, which were less pronounced at 12 months.	There were signals of promise of efficacy of EMDRp+TAU vs TAU for depression and anxiety at 6 months, which were less pronounced at 12 months.	8 EMDRp + TAU participants (27%) dropped out before session 8 due to withdrawal from the trial, engagement difficulties with EIS and the trial, or wanting to prioritize other therapies offered in EIS. 4 did not attend any sessions.
Folk 2019^44^	PP	US	22	TI-CBTp	ranged from 18–27 months	Schizophrenia spectrum disorder (58%), Mood disorder with psychotic features (42%), PTSD (58%)	Post-traumatic stress symptom severity showed trends of declining from baseline to follow-up among participants receiving stage 2 of the intervention.	Positive symptom severity was significantly higher at baseline than at 6 months and 12 months	Participants who moved to Stage 2 demonstrated a reduction in clinical symptoms over the course of 1 year of treatment, with little change in functioning or insight.	Not reported
***Lifetime Diagnosis***										
Keen 2017^45^	CS	UK	9	Integrated TF-CBT	9 months	Schizophrenia spectrum diagnosis (56%), PTSD (22%), Depressive episode with psychotic features (22%)	Overall mean scores on PDS fell from the severe range at baseline to the moderate-to-severe range at post, which was maintained at 9 month follow-up.	For delusional beliefs, 50% showed reliable improvement post therapy and at 43% at 9 month follow-up. For voices, only 25% showed reliable improvement post therapy and 29% at follow-up.	Mean scores on depression decreased from the severe range on the BDI-II (n = 5) and DASS-depression (n = 3) scales to the moderate range at post, which was maintained at follow-up. Mean scores on the BAI fell from severe to moderate at post (n = 5), which was maintained at follow-up. For those who complete the DASS-anxiety (n = 3), overall mean scores remained in the extremely severe range, but decreased to the mild range at follow-up.	No dropouts
Hardy 2022^46^	PP	UK	16	tf-CBTp	Mean of 17	Schizophrenia (37.5%), Unspecified psychosis (25%), Schizoaffective disorder (18.8%), PTSD (12.5%), Personality disorder with psychosis (6.3%)	Clinically significant change was observed in 68.8%, and post-therapy 37.5% no longer met criteria for PTSD. A worsening of PTSD symptom severity was experienced by 3 participants, which was clinically significant for 2. For these individuals, PTSD symptom severity had improved but was not sustained due to actual or threatened re-occurrence of their index trauma.	Not measured	Not measured	No dropouts
Clarke 2022^47^	MB	UK	12	ImRs	3	Mood disorder with psychotic features (50%), Paranoid schizophrenia (25%), Schizoaffective disorder (8%), Delusional disorder (8%), Bipolar disorder (8%), Psychosis NOS (8%)	83% reported reliable change on PTSD symptoms on PDS-5 at 1 week follow-up and of these 58% had clinically significant change. Three participants no longer met criteria for PTSD at follow-up.	Not measured	Not measured	No dropouts
Steel 2017^48^	RCT	UK	61	CBT-P vs TAU	12–16	In CBT group: Schizophrenia (66.7%), schizoaffective disorder (33%)	No significant difference between groups	No significant difference between groups	No significant difference between groups on depression, anxiety, and social functioning	Not reported

*Abbreviations:* CS = case series; PP = pre-post; MBS = multiple baseline; NRC = nonrandomized controlled; WGC = within-group controlled; RCT = randomized controlled trial; US=United States, UK=United Kingdom, ND=Netherlands, AUS = Australia, NZ = New Zealand, CA = Canada; CBT-*p*=CBT intervention for PTSD, BRF = 3 session breathing retraining program, TF-CBT = Trauma focused CBT, EMDR = Eye Movement Desensitization and Reprocessing, PE = Prolonged exposure, ACT = Acceptance and Commitment Therapy, ImR = imagery rescripting, NET = narrative exposure therapy, CPT-A = cognitive processing therapy (with exposure), TI-CBTp = trauma integrated CBT for psychosis, tf-CBTp = trauma focused CBT for psychosis, TRIPP = trauma-integrated psychotherapy for psychosis, STAIR = Skills Training in Affective and Interpersonal Regulation, CRI = cognitive recovery intervention, A-iMAPS = attachment-focused imagery therapy.

**Table 2.** Description of Trauma-Focused Treatments Provided

Traditional PTSD Treatments	Integrative Treatments	Experimental Treatments
***Exposure-based interventions (includes exposure, imagery rescripting, or EMDR)***		
**Prolonged exposure (PE):** Consists of psychoeducation, breathing and progressive muscle relaxation, imaginal exposure to the trauma narrative and in-vivo exposures based on a fear hierarchy^33^	**EMDRp:** Consists of the traditional 8 phase protocol, but has psychosis-specific adaptations including enhanced focus on psychoeducation and grounding; beginning work around trauma symptoms that do not reach diagnostic threshold to familiarize clients with the model prior to targeting more complex experiences; and consideration of the traumatic impact of the psychotic episode, links between trauma and psychosis symptoms, and the contribution of life experiences on maladaptive beliefs about psychotic experiences^51^	**Brief 3 session imagery rescripting (ImRs):** Involves targeting beliefs linked to a trauma memory by transforming images associated with the memory^47^
**Eye Movement Desensitization and Reprocessing (EMDR):** Consists of an 8 phase protocol that involves developing a case conceptualization and processing memories with eye movements applied as a dual-attention stimulus^42^	**Trauma-Integrated CBT for Psychosis (TI-CBTp):** Combines cognitive behavioral therapy for psychosis with empirically supported treatments for trauma, primarily trauma-focused cognitive behavioral therapy (TF-CBT) within a coordinated specialty care model for early psychosis.^44^	
**Narrative exposure therapy (NET):** Involves the creation of a timeline with significant life events, including traumatic and positive events^34^	**Trauma-Focused CBT for Psychosis (tf-CBTp):** Combines cognitive behavioral therapy for psychosis and trauma focused CBT and consists of 1) assessment, formulation, and psychoeducation 2) contextualizing memories through the use of exposure, imagery techniques or cognitive techniques 3) focusing on emotion regulation and belief systems and relapse prevention^45,46^	
**Cognitive processing therapy (CPT) + A:** Notably, there are 2 versions of the CPT protocol, one including a written narrative component (CPT + A) and one without (CPT-C). The treatment in this study included the written component, psychoeducation, emotional processing of the trauma, cognitive restructuring of trauma related stuck points.^35^ Adaptations included having participants complete homework prior to session if unable to complete outside and having one session per week instead of two.^35^	**Exposure-based Cognitive Behavioral Therapy for PTSD:** Consists of group therapy (psychoeducation, anxiety management, social skills and anger management) and individual therapy sessions (exposure therapy).^32^	
***Non-exposure based interventions***		
**Skills Training in Affective and Interpersonal Regulation (STAIR):** A group-based treatment focusing on establishing trust, safety, emotion regulation, and behavioral responses to trauma triggers^37^	**CBT intervention for PTSD (CBT-P):** Consists of psychoeducation, breathing retraining, and cognitive restructuring^31^	**Trauma-integrated psychotherapy for psychosis (TRIPP):** Consists of assessment and treatment of safety related concerns such as suicidality, self-harm, and substance use; psychoeducation about trauma; development of a timeline of major events and symptoms; and formulation of a collaborative conceptualization regarding trauma and symptom development^28^ **Acceptance and commitment therapy (ACT):** Targets experiential avoidance, lack of flexibility, and persistence in pursuing values^29^ **3 session breathing retraining program (BRF):** Consists of breathing retraining and psychoeducation facilitated via a video^39^
