# Supplementary material for: Trauma-Focused Treatment in Early and Lifetime Psychosis: A Scoping Review
Source: Schizophr Bull. Author manuscript; Available in PMC 2026 Jan 24. (PMC12831623; doi:10.1093/schbul/sbaf202)
Supplement: supplement [file NIHMS2132078-supplement-supplement.docx]

**Supplement**

**Table 1S. Preferred reporting items for systematic reviews and meta-analyses extension for scoping reviews (PRISMA-ScR) checklist**

| **SECTION** | **ITEM** | **PRISMA-ScR CHECKLIST ITEM** | **REPORTED ON PAGE #** |
| --- | --- | --- | --- |
| **TITLE** | | | |
| Title | 1 | Identify the report as a scoping review. | Pg.1 |
| **ABSTRACT** | | | |
| Structured summary | 2 | Provide a structured summary that includes (as applicable): background, objectives, eligibility criteria, sources of evidence, charting methods, results, and conclusions that relate to the review questions and objectives. | Pg. 2 |
| **INTRODUCTION** | | | |
| Rationale | 3 | Describe the rationale for the review in the context of what is already known. Explain why the review questions/objectives lend themselves to a scoping review approach. | Pg.3-4 |
| Objectives | 4 | Provide an explicit statement of the questions and objectives being addressed with reference to their key elements (e.g., population or participants, concepts, and context) or other relevant key elements used to conceptualize the review questions and/or objectives. | Pg.4 |
| **METHODS** | | | |
| Protocol and registration | 5 | Indicate whether a review protocol exists; state if and where it can be accessed (e.g., a Web address); and if available, provide registration information, including the registration number. | Pg.5 |
| Eligibility criteria | 6 | Specify characteristics of the sources of evidence used as eligibility criteria (e.g., years considered, language, and publication status), and provide a rationale. | Pg. 5 |
| Information sources* | 7 | Describe all information sources in the search (e.g., databases with dates of coverage and contact with authors to identify additional sources), as well as the date the most recent search was executed. | Pg.5 |
| Search | 8 | Present the full electronic search strategy for at least 1 database, including any limits used, such that it could be repeated. | Pg. 5 |
| Selection of sources of evidence† | 9 | State the process for selecting sources of evidence (i.e., screening and eligibility) included in the scoping review. | Pg.5 |
| Data charting process‡ | 10 | Describe the methods of charting data from the included sources of evidence (e.g., calibrated forms or forms that have been tested by the team before their use, and whether data charting was done independently or in duplicate) and any processes for obtaining and confirming data from investigators. | Pg.5 |
| Data items | 11 | List and define all variables for which data were sought and any assumptions and simplifications made. | Pg.5 |
| Critical appraisal of individual sources of evidence§ | 12 | If done, provide a rationale for conducting a critical appraisal of included sources of evidence; describe the methods used and how this information was used in any data synthesis (if appropriate). | Pg. 6 |
| Synthesis of results | 13 | Describe the methods of handling and summarizing the data that were charted. | Pg. 5 |
| **RESULTS** | | | |
| Selection of sources of evidence | 14 | Give numbers of sources of evidence screened, assessed for eligibility, and included in the review, with reasons for exclusions at each stage, ideally using a flow diagram. | Pg. 6 and Figure 1 |
| Characteristics of sources of evidence | 15 | For each source of evidence, present characteristics for which data were charted and provide the citations. | Pg. 6 and Table 1 |
| Critical appraisal within sources of evidence | 16 | If done, present data on critical appraisal of included sources of evidence (see item 12). | Supplement |
| Results of individual sources of evidence | 17 | For each included source of evidence, present the relevant data that were charted that relate to the review questions and objectives. | Pg.7-12 and Table 1 |
| Synthesis of results | 18 | Summarize and/or present the charting results as they relate to the review questions and objectives. | Pg. 6-14 |
| **DISCUSSION** | | | |
| Summary of evidence | 19 | Summarize the main results (including an overview of concepts, themes, and types of evidence available), link to the review questions and objectives, and consider the relevance to key groups. | Pg. 14 |
| Limitations | 20 | Discuss the limitations of the scoping review process. | Pg. 16 |
| Conclusions | 21 | Provide a general interpretation of the results with respect to the review questions and objectives, as well as potential implications and/or next steps. | Pg. 17 |
| **FUNDING** | | | |
| Funding | 22 | Describe sources of funding for the included sources of evidence, as well as sources of funding for the scoping review. Describe the role of the funders of the scoping review. | Pg. 1 |

JBI = Joanna Briggs Institute; PRISMA-ScR = Preferred Reporting Items for Systematic reviews and Meta-Analyses extension for Scoping Reviews.

* Where *sources of evidence* (see second footnote) are compiled from, such as bibliographic databases, social media platforms, and Web sites.

† A more inclusive/heterogeneous term used to account for the different types of evidence or data sources (e.g., quantitative and/or qualitative research, expert opinion, and policy documents) that may be eligible in a scoping review as opposed to only studies. This is not to be confused with *information sources* (see first footnote).

‡ The frameworks by Arksey and O’Malley (6) and Levac and colleagues (7) and the JBI guidance (4, 5) refer to the process of data extraction in a scoping review as data charting*.*

§ The process of systematically examining research evidence to assess its validity, results, and relevance before using it to inform a decision. This term is used for items 12 and 19 instead of "risk of bias" (which is more applicable to systematic reviews of interventions) to include and acknowledge the various sources of evidence that may be used in a scoping review (e.g., quantitative and/or qualitative research, expert opinion, and policy document).

*From:* Tricco AC, Lillie E, Zarin W, O'Brien KK, Colquhoun H, Levac D, et al. PRISMA Extension for Scoping Reviews (PRISMAScR): Checklist and Explanation. Ann Intern Med. 2018;169:467–473. [doi: 10.7326/M18-0850](http://annals.org/aim/fullarticle/2700389/prisma-extension-scoping-reviews-prisma-scr-checklist-explanation).

**Table 2S. MMAT quality assessment of studies included in review**

| **Randomized Controlled Trials** | | | | | | |
| --- | --- | --- | --- | --- | --- | --- |
| **Study** | **MMAT Questions** | | | | | **Total score (%)** |
|  | **2.1. Is randomization appropriately performed?** | **2.2. Are the groups comparable at baseline?** | **2.3. Are there complete outcome data?** | **2.4. Are outcome assessors blinded to the intervention?** | **2.5 Did the participants adhere to the assigned intervention?** |  |
| Varese et al. 2024 | Yes | Yes | Yes | Yes | No | 80 |
| Steel et al. 2017 | Yes | No | Yes | Yes | Yes | 80 |
| Every-Palmer et al. 2024 | No | Yes | Yes | Yes | Yes | 80 |
| van den Berg et al. 2015 | Yes | Yes | Yes | Yes | Yes | 100 |
| Mueser et al. 2015 | Yes | No | Yes | Can't tell | Yes | 60 |
| Mueser et al. 2008 | Yes | Yes | No | Yes | Yes | 80 |
| **Non-Randomized Studies** | | | | | | |
| **Study** | **MMAT Questions** | | | | | **Total score (%)** |
|  | **3.1. Are the participants representative of the target population?** | **3.2. Are measurements appropriate regarding both the outcome and intervention (or exposure)?** | **3.3. Are there complete outcome data?** | **3.4. Are the confounders accounted for in the design and analysis?** | **3.5. During the study period, is the intervention administered (or exposure occurred) as intended?** |  |
| de Bont et al. 2013 | Yes | Yes | Yes | Yes | Yes | 100 |
| Folk et al. 2019 | Yes | Yes | No | Yes | Can't tell | 60 |
| Trappler & Newville 2007 | Yes | Yes | Can't tell | No | Can't tell | 40 |
| Mauritz et al. 2021 | No | Yes | Yes | Can't tell | Yes | 60 |
| van den Berg et al. 2012 | Yes | Yes | Yes | No | Yes | 80 |
| Grubaugh et al. 2017 | Yes | Yes | Yes | Yes | Yes | 100 |
| Rosenberg et al.2004 | No | Yes | No | No | Yes | 40 |
| Lu et al. 2009 | No | Yes | Yes | Can't tell | Yes | 60 |
| Frueh et al. 2009 | Yes | Yes | No | Yes | Yes | 80 |
| Nishith et al. 2024 | No | Yes | No | No | Can't tell | 20 |
| **Quantitative Descriptive Studies** | | | | | | |
| **Study** | **MMAT Questions** | | | | | **Total score (%)** |
|  | **4.1. Is the sampling strategy relevant to address the research question?** | **4.2. Is the sample representative of the target population?** | **4.3. Are the measurements appropriate?** | **4.4. Is the risk of nonresponse bias low?** | **4.5. Is the statistical analysis appropriate to answer the research question?** |  |
| Jansen et al. 2017 | Can't tell | No | Yes | Yes | Yes | 60 |
| Clarke et al. 2022 | Yes | Yes | Yes | Yes | Yes | 100 |
| Keen et al. 2017 | Yes | Yes | Yes | No | Yes | 80 |
| **Mixed Methods Studies** | | | | | | |
| **Study** | **MMAT Questions** | | | | | **Total score (%)** |
|  | **5.1. Is there an adequate rationale for using a mixed methods design to address the research question?** | **5.2. Are the different components of the study effectively integrated to answer the research question?** | **5.3. Are the outputs of the integration of qualitative and quantitative components adequately interpreted?** | **5.4. Are divergences and inconsistencies between quantitative and qualitative results adequately addressed?** | **5.5. Do the different components of the study adhere to the quality criteria of each tradition of the methods involved?** |  |
| Tong et al. 2017 | Can’t tell | Yes | Yes | Yes | Yes | 80 |
| Hardy et al. 2022 | Yes | Yes | Yes | Yes | Yes | 100 |

Note. MMAT total scores are color-coded with low quality scores (0-20%) in red, medium scores (40-60%) in orange, and high quality (80-100%) in green.

**List of included studies**

1. Varese F, Sellwood W, Pulford D, et al. Trauma-focused therapy in early psychosis: Results of a feasibility randomized controlled trial of EMDR for psychosis (EMDRp) in early intervention settings. *Psychol Med*. 2024;54(5):874-885. doi:10.1017/S0033291723002532

2. Folk, Tully, Blacker, et al. Uncharted waters: Treating trauma symptoms in the context of early psychosis. *J Clin Med*. 2019;8(9):1456. doi:10.3390/jcm8091456

3. Tong J, Simpson K, Alvarez-Jimenez M, Bendall S. Distress, Psychotic Symptom Exacerbation, and Relief in Reaction to Talking about Trauma in the Context of Beneficial Trauma Therapy: Perspectives from Young People with Post-Traumatic Stress Disorder and First Episode Psychosis. *Behav Cogn Psychother*. 2017;45(6):561-576. doi:10.1017/S1352465817000236

4. Jansen JE, Morris EMJ. Acceptance and Commitment Therapy for Posttraumatic Stress Disorder in Early Psychosis: A Case Series. *Cogn Behav Pract*. 2017;24(2):187-199. doi:10.1016/j.cbpra.2016.04.003

5. Steel C, Hardy A, Smith B, et al. Cognitive–behaviour therapy for post-traumatic stress in schizophrenia. A randomized controlled trial. *Psychol Med*. 2017;47(1):43-51. doi:10.1017/s0033291716002117

6. Every-Palmer S, Flewett T, Dean S, et al. Eye movement desensitization and reprocessing (EMDR) therapy compared to usual treatment for posttraumatic stress disorder in adults with psychosis in forensic settings: Randomized controlled trial. *Psychol Trauma Theory Res Pract Policy*. 2024;16(Suppl 3):S555-S560. doi:10.1037/tra0001643

7. Trappler B, Newville H. Trauma healing via cognitive behavior therapy in chronically hospitalized patients. *Psychiatr Q*. 2007;78(4):317-325. doi:10.1007/s11126-007-9049-8

8. Mauritz MW, Van Gaal BGI, Goossens PJJ, Jongedijk RA, Vermeulen H. Treating patients with severe mental illness with narrative exposure therapy for comorbid post-traumatic stress disorder. *BJPsych Open*. 2021;7(1). doi:10.1192/bjo.2020.124

9. Van Den Berg DPG, De Bont PAJM, Van Der Vleugel BM, et al. Prolonged exposure vs eye movement desensitization and reprocessing vs waiting list for posttraumatic stress disorder in patients with a psychotic disorder: A randomized clinical trial. *JAMA Psychiatry*. 2015;72(3):259. doi:10.1001/jamapsychiatry.2014.2637

10. Mueser KT, Gottlieb JD, Xie H, et al. Evaluation of cognitive restructuring for post-traumatic stress disorder in people with severe mental illness. *Br J Psychiatry*. 2015;206(6):501-508. doi:10.1192/bjp.bp.114.147926

11. Van Den Berg DPG, Van Der Gaag M. Treating trauma in psychosis with EMDR: A pilot study. *J Behav Ther Exp Psychiatry*. 2012;43(1):664-671. doi:10.1016/j.jbtep.2011.09.011

12. Hardy A, Good S, Dix J, Longden E. “It hurt but it helped”: A mixed methods audit of the implementation of trauma- focused cognitive-behavioral therapy for psychosis. *Front Psychiatry*. 2022;13:946615. doi:10.3389/fpsyt.2022.946615

13. Clarke R, Kelly R, Hardy A. A randomised multiple baseline case series of a novel imagery rescripting protocol for intrusive trauma memories in people with psychosis. *J Behav Ther Exp Psychiatry*. 2022;75:101699. doi:10.1016/j.jbtep.2021.101699

14. Grubaugh AL, Veronee K, Ellis C, Brown W, Knapp RG. Feasibility and Efficacy of Prolonged Exposure for PTSD among Individuals with a Psychotic Spectrum Disorder. *Front Psychol*. 2017;8:977. doi:10.3389/fpsyg.2017.00977

15. de Bont PAJM, van Minnen A, de Jongh A. Treating PTSD in Patients With Psychosis: A Within-Group Controlled Feasibility Study Examining the Efficacy and Safety of Evidence-Based PE and EMDR Protocols. *Behav Ther*. 2013;44(4):717-730. doi:10.1016/j.beth.2013.07.002

16. Rosenberg SD, Mueser KT, Jankowski MK, Salyers MP, Acker K. Cognitive-behavioral treatment of PTSD in severe mental illness: Results of a pilot study. *Am J Psychiatr Rehabil*. 2004;7:171-186. doi:10.1080=15487760490476200

17. Lu W, Fite R, Kim E, et al. Cognitive-behavioral treatment of PTSD in severe mental illness: Pilot study replication in an ethnically diverse population. *Am J Psychiatr Rehabil*. 2009;12(1):73-91. doi:10.1080/15487760802615863

18. Mueser KT, Rosenberg SD, Xie H, et al. A randomized controlled trial of cognitive-behavioral treatment for posttraumatic stress disorder in severe mental illness. *J Consult Clin Psychol*. 2008;76(2):259-271. doi:10.1037/0022-006X.76.2.259

19. Frueh C, Grubaugh AL, Cusack KJ, Kimble MO, Elhai JD, Knapp RG. Exposure-based cognitive-behavioral treatment of PTSD in adults with schizophrenia or schizoaffective disorder: A pilot study. *J Anxiety Disord*. 2009;23(5):665-675. doi:10.1016/j.janxdis.2009.02.005

20. Keen N, Hunter ECM, Peters E. Integrated Trauma-Focused Cognitive-Behavioural Therapy for Post-traumatic Stress and Psychotic Symptoms: A Case-Series Study Using Imaginal Reprocessing Strategies. *Front Psychiatry*. 2017;8:92. doi:10.3389/fpsyt.2017.00092

21. Nishith P, Morse G, Dell NA. Effectiveness of Cognitive Processing Therapy for PTSD in serious mental illness. *J Behav Cogn Ther*. 2024;34(1):100486. doi:10.1016/j.jbct.2024.100486
